# Effect of Elastic Resistance on Exercise Intensity and User Satisfaction While Playing the Active Video Game BoxVR in Immersive Virtual Reality: Empirical Study

**DOI:** 10.2196/58411

**Published:** 2024-07-16

**Authors:** Jacek Polechoński, Alan Przepiórzyński, Piotr Polechoński, Rajmund Tomik

**Affiliations:** 1 Institute of Sport Sciences, Academy of Physical Education in Katowice, Katowice, Poland; 2 Student Scientific Circle of Physical Activity and Tourism in Virtual Reality, Academy of Physical Education in Katowice, Katowice, Poland; 3 Department of Health-Related Physical Activity and Tourism, Academy of Physical Education in Katowice, Katowice, Poland

**Keywords:** virtual reality, VR, game, gaming, immersive, immersion, health-related physical activity, physical activity, exercise, active video games, attractiveness, enjoyment scale, enjoyment, serious games, elastic resistance, resistance

## Abstract

**Background:**

One of the main contemporary forms of physical activity (PA) involves exercises and games in an immersive virtual reality (VR) environment, which allows the user to practice various forms of PA in a small space. Unfortunately, most of the currently available VR games and workout applications are mostly based on upper body movements, especially the arms, which do not guarantee sufficiently high exercise intensity and health benefits. Therefore, it is worth seeking solutions to help increase the exercise load during PA in VR.

**Objective:**

The main aim of this study was to evaluate the effect of elastic arm resistance in the form of latex resistance bands of different elasticity levels on the intensity of students’ PA while playing the BoxVR game. We further assessed the satisfaction of this form of exercise and its associations with PA intensity.

**Methods:**

A total of 21 healthy and physically fit men (mean age 22.5, SD 2.0 years) were included in the study. The tests consisted of 3 10-minute games. One game was run with no load and the other two were run with 1.5-meter latex resistance bands (low and high resistance). The order of the tests was randomized and the participants rested for 20 minutes after each exercise. Exercise intensity was estimated using objective (heart rate monitoring) and subjective (Borg scale) methods. The Physical Activity Enjoyment Scale was used to assess satisfaction with the PA. The effect of elastic resistance on exercise intensity and user enjoyment was estimated using ANOVA for repeated measures.

**Results:**

The ANOVA results indicated that incorporation of elastic resistance caused a significant change (*F*_2,40_=20.235, *P*<.001; η²p=0.503) in the intensity of PA in VR, which was low while playing without resistance and then increased to a moderate level with additional resistance. The use of elastic bands also changed participants’ perceptions of the enjoyment of exercise in VR (*F*_2,40_=9.259, *P*<.001; η²p=0.316). The students rated their satisfaction with PA in VR on a 7-point scale highly and similarly when exercising without an upper limb load (mean 6.19, SD 0.61) and with slight elastic resistance (mean 6.17, SD 0.66), whereas their satisfaction declined significantly (mean 5.66, SD 0.94) when incorporating a higher load.

**Conclusions:**

The intensity of PA among students playing the BoxVR game is at a relatively low level. With the added resistance of elastic bands attached to the upper limbs, the intensity of the exercise increased to a moderate level, as recommended for obtaining health benefits. Participants rated the enjoyment of PA in VR highly. The use of slight elastic resistance did not negatively affect satisfaction with the BoxVR game, although user satisfaction declined with a higher load. Further research should be undertaken to increase the effectiveness of exercise in VR so that regular users can enjoy the health benefits.

## Introduction

The past few years have seen a rapid development of technologies related to immersive virtual reality (VR). With immersive VR, the user is cut off from the visual and auditory stimuli of the surrounding reality and instead receives artificially produced images, sounds, and even tactile sensations using information technology, which is finding increasing applications in various areas of human life. In particular, VR is increasingly used for physical activity (PA) and in the development and diagnosis of physical fitness parameters. VR applications are being developed to shape and assess motor skills [1-5], research on motion analysis in VR is now actively carried out [6,7], and active virtual reality games (AVRGs) are becoming increasingly popular [8]. Feedback from users indicates the attractiveness of AVRGs, making them competitive with conventional forms of PA [9-11]. Some reports have also shown that VR can offer greater flow for PA than a similar form of exercise in the real world [12]. The great potential of AVRGs is also linked to the fact that these types of applications enable practicing different forms of exercise in a small space at home. However, one of the main limitations of VR technology in the context of its use for PA is that most of the exercises performed in the virtual environment are primarily based on movements of the upper body as the sensors placed in the VR headsets allow for tracking movements of the head and possibly the torso, while the movement sensors located in the controllers allow for tracking arm movements. Although some trainers currently work with VR headsets, such as virtual treadmills, flight simulators, and cycle ergometers, these are relatively expensive and take up space in living areas. Since PA performed in VR is mainly based on upper limb movements, there is a concern that this type of exercise may be characterized by relatively low intensity. Consequently, physical efforts practiced in a virtual environment may not be effective in terms of potential health benefits. According to World Health Organization (WHO) recommendations, PA should be characterized by moderate to high intensity to obtain health benefits [13]. Therefore, solutions should be sought to increase the body’s workload when practicing PA in VR.

The first related studies are already being carried out. One of the proposals to increase the intensity of physical exercise in VR is to use an additional load in the form of handheld weights (HHWs). Based on the experiments carried out to date, this type of solution can be effective [10]. An alternative solution to Velcro-fastened weights could be elastic resistance in the form of rubber bands attached to the distal parts of the upper limbs. Indeed, resistance bands have been widely used in fitness classes and various sports to improve the effectiveness of training [14-17]. Such a solution may be used provided there is effective stretching of the elastic bands during arm movements in VR. Applications that meet this condition include the popular AVRGs based on boxing techniques. With appropriately fastened straps, boxing movements can be performed with elastic resistance, which should potentially increase the intensity of this type of PA. However, the specific amount of resistance that should be applied to increase the effectiveness of the exercise while not causing discomfort due to the excessive load remains unclear, as this could negatively affect the attractiveness of PA perceived by users.

Therefore, the main aim of this study was to evaluate the effect of elastic resistance in the form of latex resistance bands with different elastic properties on exercise intensity in young and physically fit adults while playing the popular AVRG game BoxVR [18]. The results obtained were related to the WHO health recommendations for PA. This study further assessed the attractiveness of such a form of exercise and the relationships between the use of elastic resistance and user satisfaction. It was hypothesized that the use of resistance bands would significantly increase the participants’ exercise intensity and would not significantly affect their assessment of satisfaction with playing the AVRG.

## Methods

### Participants

The study involved 21 healthy and physically fit men studying at the Academy of Physical Education in Katowice, Poland (mean age 22.5, SD 2.0 years; mean body height 181.6, SD 7.3 centimeters; mean body weight 79.5, SD 11.0 kilograms). People with motion sickness, sensitivity to flashing lights, epileptic seizures, and balance disorders were excluded from the study. The research was carried out at the Jerzy Kukuczka Academy of Physical Education in Katowice, Poland, at a certified Laboratory of Research on Pro-Health Physical Activity (PN-EN ISO 9001:2015, certificate validity: 7.12.2021‐16.12.2024).

### Ethical Considerations

The study was conducted according to the guidelines of the Declaration of Helsinki, and was reviewed and approved by the Research Ethics Committee of the Jerzy Kukuczka Academy of Physical Education in Katowice (protocols: 9/2018; KB/27/2022). All participants took part in the study voluntarily and could discontinue their participation at any time. All participants were familiarized in detail with the purpose of the study, safety rules, the use of the VR equipment, and the course of the study. In addition, a written informed consent form was provided to all eligible study participants. The test results were secured in accordance with the security procedures in force at the Laboratory of Research on Pro-Health Physical Activity.

### Research Tools and Procedures

**Figure 1.**
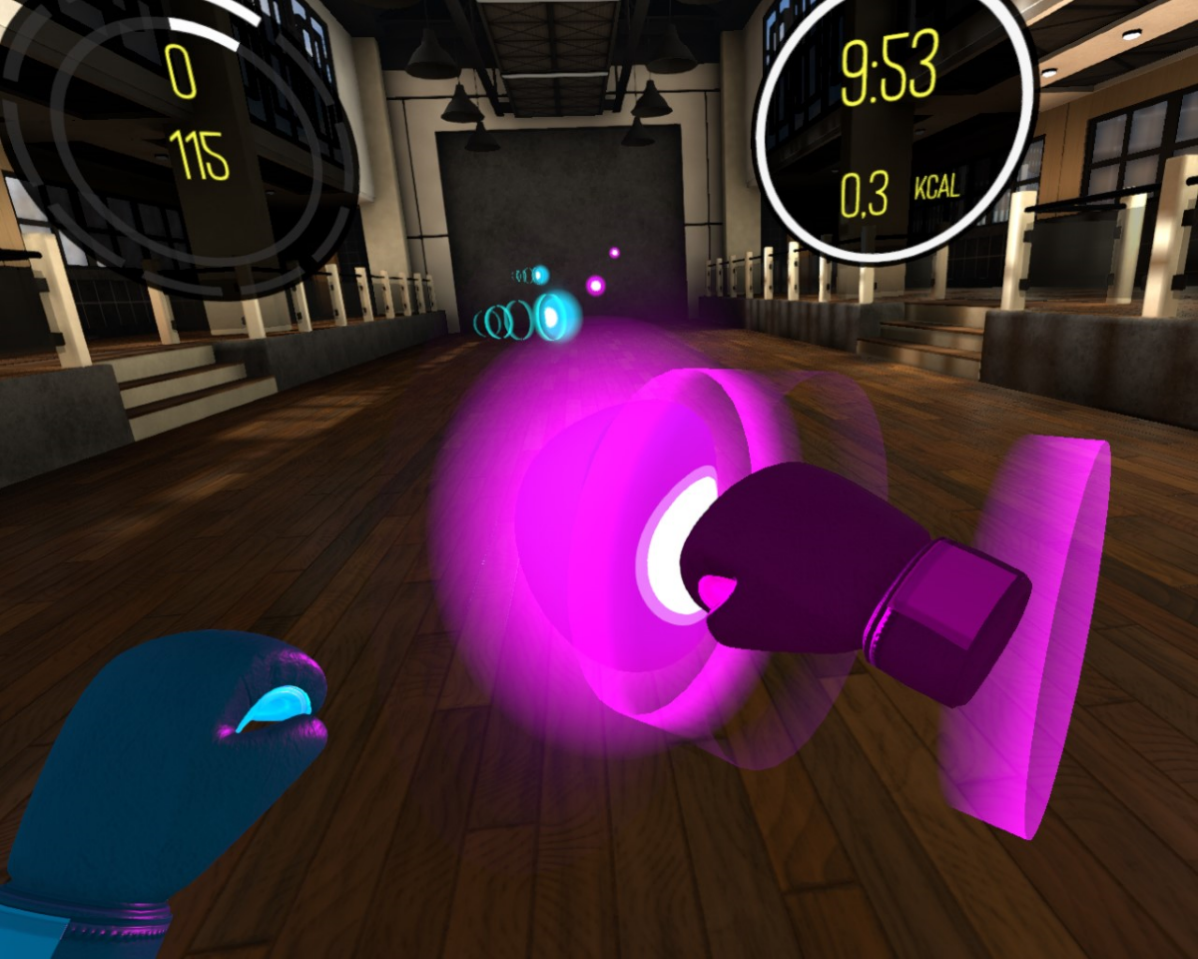
Screenshot showing a view of the BoxVR game environment from the user’s perspective.

An HTC Vive (HTC Corporation, New Taipei, Taiwan) kit was used for immersive VR, consisting of a headset, two base stations, two controllers, and a computer. The HTC Vive set is one of the popular VR systems on the market, which is characterized by high visual quality and allows for realistic VR experiences. This system was selected for this research since the motion-tracking system in HTC Vive is very precise and accurate, which allows the user to move smoothly and naturally in the virtual environment. Owing to a set of sensors and controllers, users can freely explore the virtual world. The BoxVR application was used with several training programs of varying difficulty. These programs involve boxing routines combined with music, reminiscent of shadow boxing. The game is based on basic boxing punches (ie, straight, hook, and undercut), which are performed on virtual objects coming from the depths of the room to the rhythm of the music in various combinations. There are also shapes the user has to avoid or block. The user hits targets with their hands, which they perceive in the virtual environment as boxing gloves in two colors: blue (left hand) and pink (right hand). An illustration of the playing environment is provided in Figure 1. To obtain a high score in the game, the user has to execute the punches correctly and hit the targets moving toward them that correspond to the appropriate glove color. The user scores points for every correct response. In the case of a series of several or more accurate hits, the score is further multiplied by an appropriate multiplier. Information on the number of points scored and the duration of the game is displayed on virtual screens in front of the user.

The systsem has a panel that allows the user to select game modes with different durations, levels of difficulty, and nature of exercise. For the purposes of the study, mode “seventeen” was selected lasting 10 minutes, set at medium difficulty and with the no squat option. This mode was considered to be optimal for physically fit young adults. The squat option was disabled because the aim of the study was to assess the impact of the elastic resistance of the arms on the intensity of physical exercise; therefore, additional lower limb exercises would be a factor that could complicate the interpretation of the study results. It should be noted that many active video games (AVGs) are based solely on arm and torso movements. Before the study, participants were familiarized with the use of the application and took part in a short (2-minute) no-load trial.

**Figure 2.**
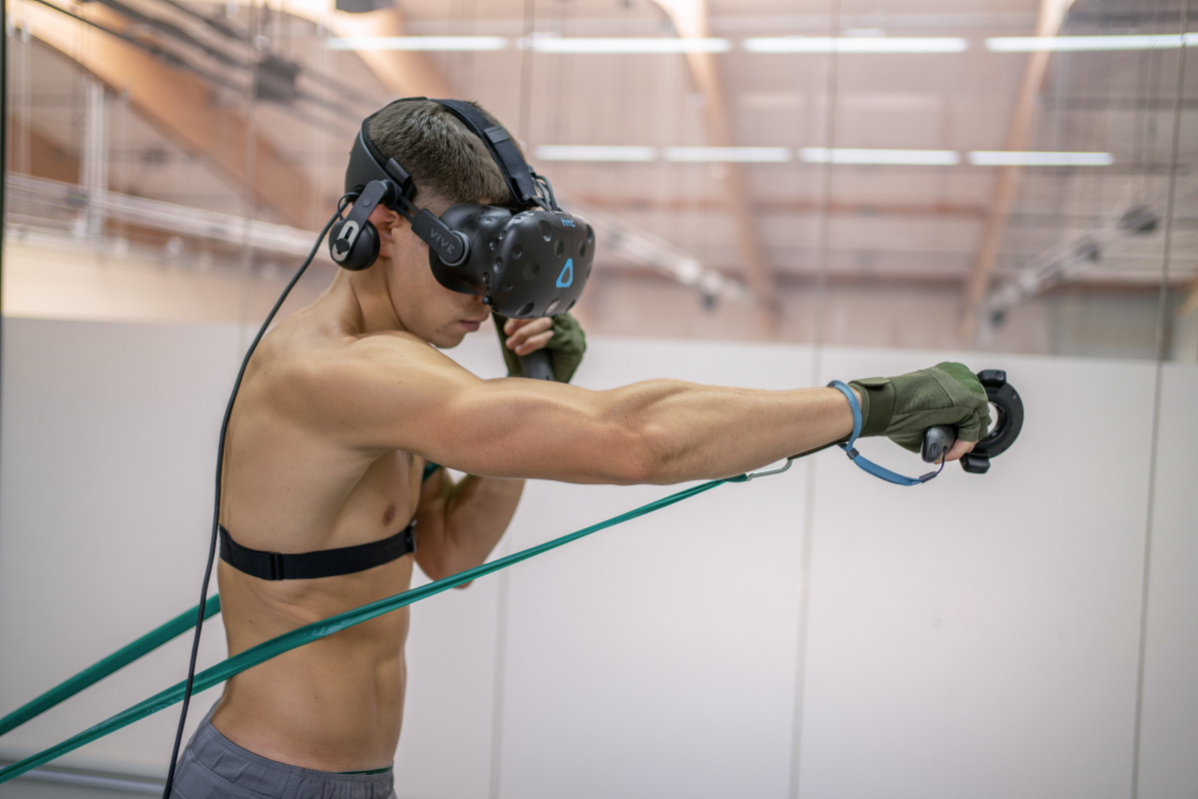
A participant during a resistance exercise with the green band.

The tests consisted of 3 10-minute games. One game was run with no load and the other two were run with 1.5-meter latex resistance bands, including green (low resistance) and silver (high resistance) bands. The elasticity characteristics of the resistance bands provided by the manufacturer (Thera-Band) are presented in Table 1. The bands were attached to the ground on one side and to tactical gloves (M-Tac) on the other. When starting the exercise with the bands, users held their hands in a guard position and were positioned at an appropriate distance from the point of attachment ensuring that the bands were taut but not stretched. When the punches were performed, the bands stretched, causing resistance (Figure 2). The order of the tests was randomized, and the participant rested for 20 minutes after each exercise before starting the next game.

While playing the VR game, participants’ heart rates were monitored using a Vantage V heart rate monitor (Polar Electro Oy, Kempele, Finland) coupled with a chest strap (Polar H10). Based on the average exercise heart rate (HR_ave_), the PA intensity was estimated as the average percentage of maximum heart rate (% HR_max_). The HR_max_ value was first estimated from the formula 208 – 0.7 × age (years) [19]. The results obtained were compared to the PA intensity standards recommended by the American College of Sports Medicine [20]. According to this classification, it is assumed that during low-intensity exercises, HR_ave_<64% of HR_max_, high-intensity PA occurs when HR_ave_≥77% of HR_max_, and moderate exercise is defined in the condition of HR_ave_≥64% of HR_max_ with less than 77% of HR_max_. Furthermore, the average absolute duration of PA (in seconds) was estimated for the following exercise intensity zones: 0, less than 50% HR_max_; 1, 50%‐59% HR_max_; 2, 60%‐69% HR_max_; 3, 70%‐79% HR_max_; 4, 80%‐89% HR_max_; and 5, ≥90% HR_max_. These intensity zones were selected because they are used to report the results in the software of the heart rate monitor (Vantage V) used in the study.

At the end of each test, the participants also self-assessed their perceived exertion using the Borg Rating of Perceived Exertion (RPE), which ranges from 6 to 20 [21,22]. According to this scale, a score of 10-11 indicates low-intensity exercise, a score of 12-13 indicates moderate-intensity exercise, and a score of 14‐16 indicates high-intensity exercise [23]. The RPE scores were compared with the objective measurements to determine the correlation of PA intensity with the subjective perceptions of the participants.

Subsequently, the participants assessed their satisfaction with PA in VR using the long version of the Physical Activity Enjoyment Scale (PACES), consisting of 18 items [24], which were answered after each test on a 7-point Likert scale. The average calculated from all responses was used for analysis.

**Table 1.** Specifications of the elastic bands (Thera-Band) used during testing.

Stretch, %	Resistance, kilograms	
Green band	Silver band
25	0.9	2.3
50	1.5	3.9
75	1.9	5.0
100	2.3	6.0
125	2.6	6.9
150	3.0	7.8
175	3.3	8.6
200	3.6	9.5
225	4.0	10.5
250	4.4	11.5

### Statistical Analysis

Basic descriptive statistics (arithmetic means and SDs) were calculated. The Shapiro-Wilk test was used to assess whether the data followed a normal distribution, whereas sphericity was assessed using the Mauchley test. The effect of elastic resistance on exercise intensity was estimated using ANOVA for repeated measures or Friedman ANOVA, depending on the distribution of the data. The repeated-measures ANOVA was supplemented with Tukey posthoc tests, whereas Friedman ANOVA was followed by the Dunn posthoc test. The level of statistical significance was set at α=.05. The effect size was estimated using η²p or the Kendall coefficient (*W*). The Spearman rank correlation coefficient (*r*_S_) was used as a measure of the relationship between objective and subjective intensity measures. Statistical analyses were performed using Statistica v.13 (TIBCO Software Inc) and Jamovi v. 2.2.3.0 software.

## Results

### Exercise Intensity in VR Without Upper Limb Loading and With Elastic Resistance

**Figure 3.**
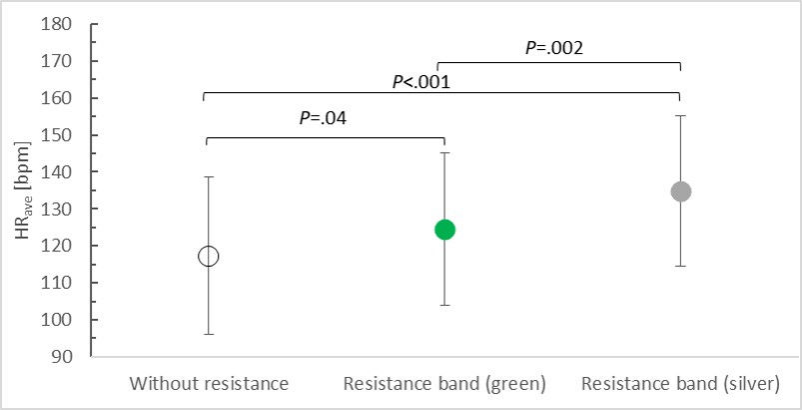
Average heart rate while playing BoxVR depending on upper limb load. bpm: beats per minute; HR_ave_: average heart rate.

Repeated-measures ANOVA showed that elastic resistance significantly affected the participants’ heart rate (*F*_2,40_=20.151, *P<*.001; η²p=0.503). When playing without external resistance, the heart rate was the lowest, with a mean of 117.33 (SD 21.21) beats per minute (bpm). The heart rate increased to a mean of 124.43 (SD 20.62) bpm during play with the green elastic band and increased further to a mean of 134.90 (SD 20.33) bpm during exercise with the silver band. Posthoc tests showed statistically significant differences between the results of all measurements taken according to the level of resistance applied (Figure 3).

**Figure 4.**
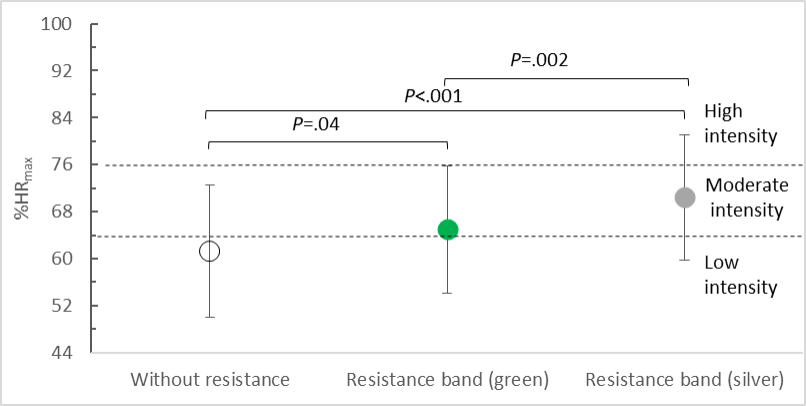
Intensity of physical exercise while playing BoxVR depending on upper limb load. % HR_max_: percentage of maximum heart rate.

Elastic resistance also resulted in a significant change (*F*_2,40_=20.235, *P*<.001; η²p=0.503) in exercise intensity as expressed by the mean %HR_max_. While playing without resistance, the intensity of physical effort was low, with a mean %HR_max_ of 61.27% (SD 11.21%). With elastic resistance, physical effort (%HR_max_) increased to a moderate level, as recommended for health benefits, for both the green (mean 64.97%, SD 10.86%) and silver (mean 70.43%, SD 10.68%) bands. Posthoc tests revealed statistically significant differences between the results of all tests according to varying levels of resistance (Figure 4).

**Figure 5.**
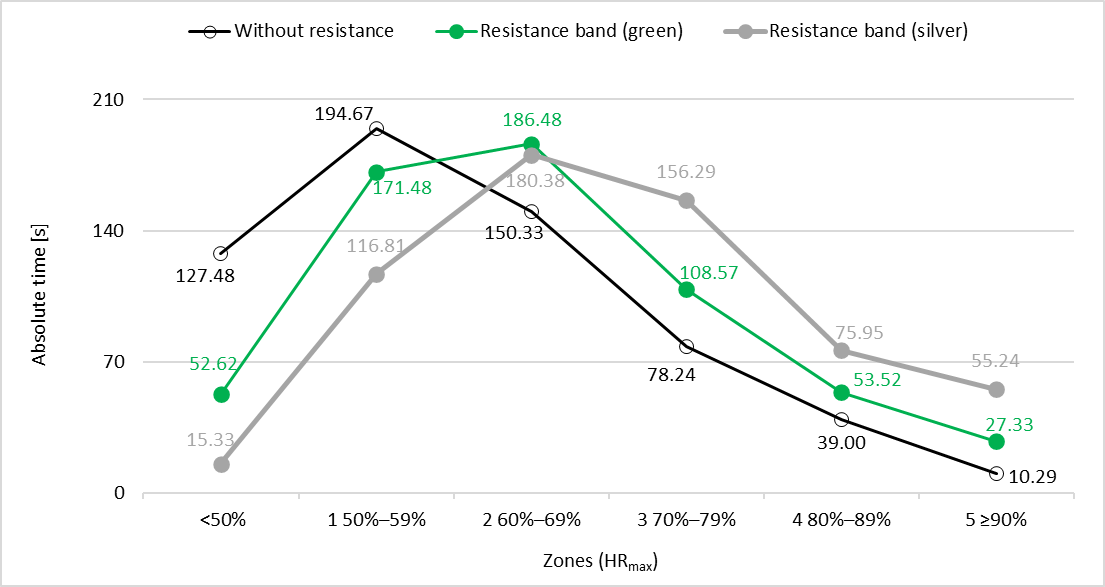
Average time spent in different heart rate zones by participants depending on upper limb load while playing BoxVR; HR_max_: maximum heart rate.

Under conditions of no external load, the participants’ heart rates lasted the longest in zone 1 (50%‐59% of HR_max_), whereas in both cases of exercise with elastic bands, the heart rates remained in zone 2 (60%‐69% of HR_max_). The Friedman ANOVA showed significant variation in results for zones: 0 (*χ*^2^_2_=14.711, *P*<.001; *W*=0.350), 3 (*χ*^2^_2_=8.954, *P*=.01; *W*=0.213), and 5 (*χ*^2^_2_=9.333, *P*=.009; *W*=0.222). No significant effect of arm loading on PA intensity was found for the other zones: 1 (*χ*^2^_2_=3.610; *P*=.17; *W*=0.086 ), 2 (*χ*^2^_2_=2.913; *P*=.33; *W*=0.052), and 4 (*χ*^2^_2_=3.360; *P*=.19; *W*=0.080) (Figure 5).

**Figure 6.**
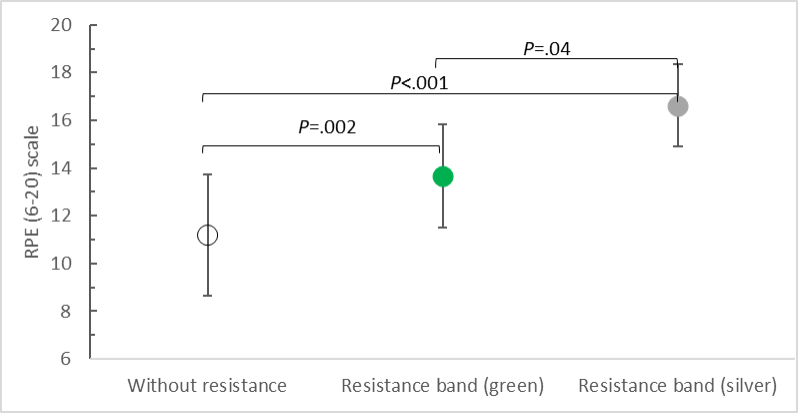
Rating of Perceived Exertion (RPE) scale versus upper limb load while playing BoxVR.

We further analyzed the exertion perceived by the users after each test based on the RPE scale (6-20). Friedman ANOVA demonstrated that elastic resistance loading significantly (*χ*^2^_2_=36.861, *P<*.001; *W*=0.878) altered users’ perceptions of exertion. The lowest fatigue was declared by those exercising without additional load. The exercise intensity in this case was rated a mean of 11.19 (SD 2.54) points. In contrast, significantly more exertion was reported for PA with elastic resistance. The mean scores for the intensity of exercise were 13.67 (SD 2.15) for the green band and 16.62 (SD 1.72) for the silver band. Posthoc tests for the pairwise comparisons of the results revealed statistically significant differences (Figure 6).

Spearman correlation analysis between subjective and objective measures of exercise intensity showed a statistically significant positive relationship between intensity evaluated based on %HR_max_ and the Borg RPE scale (6-20) for PA in VR without load (*r*_S_=0.504, *P=*.02) and with green elastic band resistance (*r*_S_=0.45, *P=*.04). No significant correlation was found for physical exercise with the silver band (*r*_S_=0.356, *P=*.11).

### Satisfaction of Study Participants With Exercise in VR

**Figure 7.**
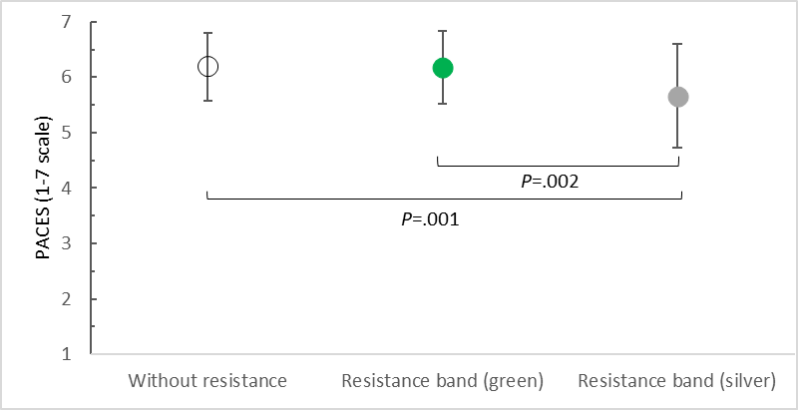
Satisfaction of participants with physical activity in virtual reality versus upper limb load. PACES: Physical Activity Enjoyment Scale.

The ANOVA of the PACES questionnaire results showed that additional elastic resistance significantly (*F*_2,40_=9.259, *P<*.001; η²p=0.316) influenced participants’ perceptions of the attractiveness of exercise in VR. Study participants rated their satisfaction with PA in VR very similarly and highly for exercise without an upper limb load (mean 6.19, SD 0.61 points) and with elastic resistance in the form of a green band (mean 6.17, SD 0.66 points). The differences between these scores were minimal and statistically insignificant. Study participants were by far the least satisfied with PA in VR with the silver band (mean 5.66, SD 0.94 points). Therefore, statistically significant differences were found between the results of the assessment of exercises without additional resistance and with the silver band *(P*<.001) and between the assessment of exercises with the green and silver bands *(P*=.002) (Figure 7).

## Discussion

### Principal Findings

This study found that the use of elastic bands while playing the AVRG BoxVR had a significant effect on exercise intensity, as shown by objective measurements and manifested by an increase in the heart rate. Furthermore, PA accompanying boxing exercises in VR, which was classified as low-intensity exercise, became moderately intense with additional resistance, and therefore became an exercise program that is considered beneficial for health according to WHO recommendations [13]. The heart rates remained in the high zones for a longer period of time during the resistance band exercises. Therefore, this type of shoulder loading during PA in VR appears to be an effective solution for increasing the intensity of physical exercise based on arm work.

The method presented in our study to increase PA intensity in VR by using resistance bands represents a novel solution. To date, weights attached to the distal part of the limbs have been used for this purpose. A recent study showed that the use of 0.5-kilogram Velcro-fastened HHWs placed on the wrists increased the intensity of PA in VR [10]. The authors found that under such upper limb loading, the PA intensity while playing the popular AVRG Beat Saber increased from low to moderate, thus becoming a healthy exercise. Similar studies were carried out using 2-kilogram ankle weights while playing an AVRG based on locomotor movements and practiced on an Omni omnidirectional treadmill (Virtuix) [11]. In this case, the additional load on the lower limbs was found to significantly increase physical exertion during virtual active entertainment. Recently, manufacturers of VR accessories have begun to view additional limb loading as a way to improve the effectiveness of exercise in a virtual environment. More recently, special controller overlays in the form of small discs have been offered for sale for users of the Oculus Quest 2 headset, which, when placed on the controllers, cause an increase in their weight to increase the intensity of exercise while using the application. As the use of resistance bands promotes increased PA intensity in arm-based VR, the use of elastic resistance offers an alternative to HHW or controller-mounted weights. However, there is currently a lack of such dedicated solutions for VR users.

The effect of elastic arm resistance on the intensity of physical exertion of users while playing BoxVR was also evidenced by the participant-reported RPE scores, which increased significantly after the use of elastic bands. Comparison of RPE reported by the participants with the objective classification of PA intensity [23] revealed that the students rated the PA without external loading as light and that with the green elastic band as moderate, which was similar to the objective assessment based on the heart rate monitor. In contrast, physical exercise performed with the silver band was rated as vigorous by the participants, indicating that the students overestimated its intensity in relation to objective measurements. This overestimation may be confirmed by the correlation analysis between subjective and objective measures of exercise intensity, showing a statistically significant relationship between RPE and %HR_max_ for PA in VR without a load and with the green elastic band resistance, while no significant relationship was found for exercise with the silver band. The exaggerated level of the subjective rating of PA during exercise in VR is somewhat puzzling, as previous studies have demonstrated that being in a VR environment reduces the intensity of perception of various stimuli (eg, pain) because VR, by stimulating different senses, distracts the immersed person from the problem [25-28]. During exercise in VR, this phenomenon, known as cognitive distraction, can alleviate the discomfort associated with hard training. The few studies on this topic published to date suggest that VR may be useful in distracting from unpleasant bodily sensations occurring during aerobic PA in children with overweight and obesity [9] and in reducing negative sensations associated with the performance of isometric exercises [29].

According to the PACES survey, study participants highly rated their satisfaction with PA in a virtual environment while playing BoxVR. Scores exceeded 6 on a 7-point scale for two measurements. Although the ANOVA of the PACES questionnaire results revealed that additional elastic resistance significantly affects the participants’ perceptions of the attractiveness of exercise in VR, study participants rated their satisfaction with PA in VR very similarly for exercise without upper limb loading and with green resistance bands; the differences found were minimal and statistically insignificant. This may indicate that the low external load on the arms does not bother users and does not cause discomfort that could reduce the enjoyment of the exercises performed in VR. This was also confirmed by the aforementioned studies using a 0.5-kilogram HHW and a 2-kilogram ankle weight [10,11]. However, our results suggest that as the elastic external load on the arms increases, there may be a reduction in user satisfaction with PA in VR. Participants in this study were the least satisfied with playing BoxVR while having to overcome the resistance of the silver band, although a score of 5.66 still seems to be relatively high. The attractiveness of PA in VR has also been assessed in other contexts [10-12,30-36], and most of these studies have indicated a high level of user satisfaction with such exercises. Because those studies assessed other forms of PA or the attractiveness of physical exercise was measured with different tools, it is difficult to compare their results with those obtained in our study. Due to the rapid development of AVRGs and training applications used in a virtual environment, further research is warranted to identify the determinants of satisfaction of people participating in PA in VR. This will help guide the further development of this new form of exercise. Notably, satisfaction is an important motivation for undertaking regular healthy PA, and how people feel when they exercise determines their future training engagement [37]. Therefore, identifying user preferences for different forms of PA in VR can increase the likelihood of the regular active use of modern technology, which should translate into health benefits.

### Limitations and Prospects

Despite these promising results, the solution we have presented has some limitations. Namely, for the user to perform the exercise with the resistance band attached to the ground, they must be looking forward and cannot move freely. Consequently, the use of such a solution is only possible for certain AVRGs. However, there is a way to address this limitation. There are wearable resistance band (WRB) systems (eg, WearBands or MASS Suit), using specially designed belts, socks, gloves, and other items of clothing to anchor elastic resistance bands connecting two or more body segments. WRB training is a new entry to the field of resistance training. With WRBs, the exerciser can move freely while performing movements with elastic resistance [38]. Despite the lack of research on exercise with WRB, it appears that this type of training system may be useful for increasing the intensity and effectiveness of exercise in VR. Testing this assumption may provide objectives for further empirical research. Currently, elastic resistance is being used in AVGs in nonimmersive VR. An example is the original pointing device created by Nintendo called Ring Fit, which works with the Ring Fit Adventure app. This is an elastic ring that can be squeezed, stretched, and moved in space, which enable controlling the movements of the virtual avatar. The few studies conducted to date have shown that Ring Fit exercises have a beneficial effect on students’ physical fitness [39], reduce lower back pain in adults [40], improve balance in older people [41], and may be a useful form of PA for children with overweight and obesity by increasing their daily energy expenditure [42].

### Conclusions

The intensity of PA among students playing the BoxVR game is at a relatively low level. With the added resistance of elastic bands attached to the upper limbs of the participants, the intensity of the exercise increased to a moderate level, as recommended for obtaining health benefits. Participants in this study highly rated the attractiveness of PA in VR. The use of slight elastic resistance did not negatively affect the satisfaction of study participants with the BoxVR game, although the satisfaction declined with a higher load.

Due to the rapid development of VR, the great popularity of games and training programs in a virtual environment, and their attractiveness to users, it is expected that more and more people will enjoy active entertainment in VR. Therefore, research should be undertaken to assess user preferences and seek solutions to increase the usefulness and effectiveness of this newly developed form of PA so that regular users can improve their physical fitness and reap the health benefits. Our study may provide guidance to VR equipment manufacturers on how to make exercise more effective while playing AVRGs based on upper limb movements. However, the validity of the considerations outlined above should be confirmed in further research using applications that allow various forms of PA to be practiced in an immersive VR environment.

## Abbreviations

%HR_max_: percentage of maximal heart rate
AVG: active video game
AVRG: active virtual reality game
bpm: beats per minute
HHW: handheld weight
HR_ave_: average heart rate
HR_max_: maximum heart rate
PA: physical activity
PACES: Physical Activity Enjoyment Scale
RPE: Rating of Perceived Exertion
VR: virtual reality
WHO: World Health Organization
WRB: wearable resistance band
